# Routine cervical screening with primary HPV testing and cytology triage protocol in a randomised setting

**DOI:** 10.1038/sj.bjc.6602799

**Published:** 2005-09-27

**Authors:** L Kotaniemi-Talonen, P Nieminen, A Anttila, M Hakama

**Affiliations:** 1 Mass Screening Registry, Finnish Cancer Registry, Liisankatu 21 B, FI-00170 Helsinki, Finland; 2 Department of Obstetrics and Gynaecology, Helsinki University Central Hospital, POB 140, FI-00029 HUS Helsinki, Finland; 3 University of Tampere, School of Public Health, FI-33014 University of Tampere, Tampere, Finland

**Keywords:** cervical cancer, screening, high-risk HPV, randomised, evaluation, public health

## Abstract

The role of high-risk human papillomavirus (hrHPV) testing in primary cervical screening has not been established. We generated a randomised evaluation design ultimately to clarify whether primary hrHPV testing implemented into routine screening can bring increase in the programme effectiveness. The aim of the present report on first-year results was to assess the cross-sectional relative validity parameters for routine hrHPV screening, in comparison with conventional screening. An equal number of women invited to routine screening was randomly allocated to primary hrHPV screening (*n*=7060) and to cytological screening (*n*=7089). In the hrHPV screening arm, after a single positive hrHPV test result, the need of colposcopy referral was determined by a cytological triage test. Compared with the conventional arm, more colposcopy referrals were made in the hrHPV screening arm (relative risk 1.51, confidence interval 95% 1.03–2.22). Specificity of the primary screening with sole hrHPV test (91.5–92.1%) was much lower than that with the cytology triage (98.7–99.3%), which was not quite as specific as screening with conventional cytology (99.2–99.6%). Compared with conventional cytology, primary screening with hrHPV test results in increased cross-sectional relative sensitivity at the level of all positive lesions at the cost of substantial loss in specificity. With cytology triage, the specificity improves to the level of conventional cytology.

On the area of cervical cancer prevention, much emphasis and hope has lately been given on human papillomavirus (HPV) vaccines being tested around the world (Koutsky *et al*, 2002; Harper *et al*, 2004; Munoz *et al*, 2004). Nevertheless, organised population-based screening is still the most effective way to control for cervical cancer (The working group of IARC, 2005). First screening programmes were based on a conventional Papanicolaou or ‘Pap’ smear analysed with light microscopy. However, with evolution of medical technology, alternative screening modalities have been developed and introduced into the routine screening in increasing numbers (Noorani *et al*, 2003; Anttila *et al*, 2004; Nieminen *et al*, 2005; The working group of IARC, 2005). Establishing the key role of certain human papillomavirus types (referred as high-risk HPVs (hrHPVs)) in causing cervical cancer has given birth to the generation of aetiology-based screening tests (Schiffman *et al*, 1993; Bosch *et al*, 1995; Jacobs *et al*, 1997; Walboomers *et al*, 1999; Lorincz, 2003).

Recently, the role of high-risk HPV testing in primary screening has been a subject of wide interest. Screening for hrHPVs is suggested to be beneficial, especially in settings where it is used to clarify the importance of cytological test result (Castle *et al*, 2002; Lorincz and Richart, 2003; Noorani *et al*, 2003; Arbyn *et al*, 2004; Wright *et al*, 2004; The working group of IARC, 2005). Instead, whether primary hrHPV testing implemented into an existing and well-functioning public-health screening programme can bring increase in the programme effectiveness is not known. To study this, the commercial hrHPV detection test, HC 2^©^, has been implemented by randomisation into the Finnish cervical cancer screening programme as the primary screening test since 2003.

The aim of the current report on first-year results is to assess the relative validity parameters, that is, cross-sectional sensitivity and specificity, for routine hrHPV screening, in comparison with conventional screening.

## MATERIALS AND METHODS

### Finnish cervical screening programme

Finnish cervical screening programme is organised and nation-wide, targeted for 30–60-year-old women. Individual municipalities are responsible for the costs and practical arrangements. The screening interval is 5 years, unless intensified (or risk group) screening is indicated by previous screening test result or reported symptoms; in this case, the screening interval is shortened to 1 year (Hakama *et al*, 1979; Anttila & Nieminen, 2000). Women belonging to the target population are identified from the national Population Registry and invited by a personal letter to free cervical screening. During the screening visit a trained nurse or midwife takes the screening sample, traditionally a cytological smear of three cervicovaginal subsamples (vaginal, cervical and endocervical), that is, a VCE smear. In addition to this, for each participant the sample-taker fills an information form of recent gynaecological history. Completed forms and fixed samples are sent to screening laboratories for further processing and analysis. Eventually, screening results are mailed from the screening laboratories to the attended women. However, if colposcopy is indicated on the basis of the screening result, the woman is generally contacted first by phone.

Primary smear analysis is performed in screening laboratories by cytotechnicians. A pathologist re-screens all the slides with any abnormalities and a small fraction of the slides classified normal. The cytology results are reported according to modified Papanicolaou classification, in which the specimen adequacy is evaluated and descriptive diagnosis given in addition to designating the cytological findings class I (normal) through to class V (malignant). Women with class III–V smears (mild, moderate and severe dyskaryosis as well as carcinoma cells in British terminology; ASC-H, LSIL, HSIL and glandular atypia in TBS 2001) are directly referred for colposcopy. Instead, class II smears (borderline changes in British terminology, reactive and ASC-US in TBS 2001) are considered essentially benign in cellular changes and, thus, they are most often recommended to control for by a new smear after 6–12 months or after the possible medicinal treatment. In addition, in most municipalities women with class II smears are invited to intensified screening after 1 year. Screening-induced colposcopies and other possible confirmatory tests are conducted mainly in regional hospitals, where the possible histologically confirmed precancerous lesions are treated and their postoperational follow-up is carried out. Women with negative histological confirmation are invited to intensified screening a year after.

For each screening episode within the invitational programme, the data on screening test results, colposcopy referrals and histological findings are sent by the screening laboratory personnel to the Mass Screening Registry of the Finnish Cancer Registry. Mass Screening Registry does not collect any information on smears from outside the organised programme.

### Study design

The routine screening target population of seven committed municipalities (Hyvinkää, Järvenpää, Kirkkonummi, Lohja, Porvoo, Tuusula, Vantaa) was individually randomised 1 : 1 to the hrHPV screening arm with primary hrHPV screening (HC 2^©^ by Digene Corporation, MD, USA) with cytology triage and to the control arm with conventional cytological testing by randomly allocating the women on the basis of their personal identifiers, while drawing the invitational information from the Population Registry files. Each identified woman was invited with a similar personal letter mailed together with a special information brochure. Individual randomisation status was not revealed in the letter, but it was registered to the Mass Screening Registry, from where it was controlled, that the randomised women would get the same statuses each time they were invited within the programme.

In the control arm, screening visit procedures were not altered from the previous routine. Instead, in the hrHPV arm two separate samples were taken per each attendee: an hrHPV test sample (HC 2^©^) for primary screening test and a VCE smear for the cytological triage test. The two spatular subsamples of the VCE smear were collected with wooden or plastic Ayre's spatulas and the endocervical subsample with the special cone-shaped cervical sampler brush of the HC 2^©^ test kit. After the VCE smear was prepared from the subsamples, the tip of the cervical sampler brush was placed into HC 2^©^ transport medium tube to bring it into hrHPV test sample, and the tube was labelled with a sticker containing the same patient information as the VCE smear. In the case that the screenee explicitly refused from the hrHPV test, only a VCE smear was taken.

All the samples were sent to the Helsinki screening laboratory of Cancer Society of Finland (CSF) within 2 weeks from the sample taking. In the laboratory smears were stained, but only smears of the conventional arm were immediately directed to analysis. The intervention arm smears were either screened or stored, depending on the primary hrHPV test result: if the hrHPV test resulted negative (ratio of the relative light units (rlu ratio) less than 1.00), the smear was stored unscreened; if positive (rlu ratio ⩾1.00), the cytological analysis was carried out. In case the primary hrHPV test sample was erroneously not taken, primary cytological analysis was carried out instead, as in the conventional arm. The few hrHPV test samples collected in the conventional arm were not analysed but destroyed.

In the conventional arm, positive cytology test result (Pap class III–V) was confirmed with colposcopy and biopsies. In the hrHPV screening arm, in case of a single positive primary screening test, the potential need of histological confirmation was assessed by the cytology triage test, that is, confirmation with colposcopy and biopsies was carried out only when cytological result was suggestive of dysplasia or cancer (Pap class III–V); instead, cytological normal to benign changes (Pap class I–II) were considered a reason for intensified screening due to current hrHPV infection and, thus, after 12 months from the initial visit a risk group invitation within the organised programme was to follow. Within the risk group screening, women with persistent hrHPV infection, indicated by repeated hrHPV positivity, will be identified and eventually referred for colposcopy (not reported here). Colposcopies of both screening arms were conducted in local hospitals for six municipalities, and for one municipality (Vantaa) they were done in the practice of CSF, located in connection with the CSF Helsinki screening laboratory. At any stage, hrHPV test results were not blinded from the laboratory and hospital personnel.

### Ethical issues

Before launching the trial, our hrHPV screening design with cytology triage protocol was accepted by the principal authorities in medico-ethical issues in Finland, the National Authority for Medicolegal Affairs and the Ethical Committee of Obstetrics and Gynecology in Hospital District of Helsinki and Uusimaa, which enabled running of the trial within the Finnish cervical screening programme, that is, launching routine screening arm with primary hrHPV testing. Also, health boards of the contacted municipalities gave their acceptance to routine hrHPV screening with the current design. Sample-takers of the participating municipalities received a half-day training for the technical procedures of HC 2^©^ sample taking and for informing the attending women of the hrHPV screening and of the implication of their individual randomisation status; the manufacturers’ representative trained for 3 days the personnel of the responsible screening laboratory for HC 2^©^ analysis.

Permission of the National Authority for Medicolegal Affairs stated, on the basis of the Act of the Medical Use of Human Organs and Tissues (2001), that as the randomised implementation of hrHPV testing to the nation-wide cervical screening programme was expected to result in a very large number of primary hrHPV tests, collecting an informed consent from each attendant was not required; instead, invited women had to be properly informed on different screening modalities prior to the screening visit, and women not willing to have an hrHPV test were to be screened with conventional method. For this, we designed a brochure containing essential information on cervical cancer epidemiology, role of HPV infection in the cancer development, purpose of cervical screening, screening visit procedures and hrHPV testing within the routine screening, which was mailed together with the personal invitation letter for the targeted women in the participating municipalities. The individual randomisation status was not given in the invitation letter, as we considered that it might affect the participation rate, but it was discussed during the screening visit to allow women to refuse from the hrHPV test.

### Statistical analysis

**Figure 1:**
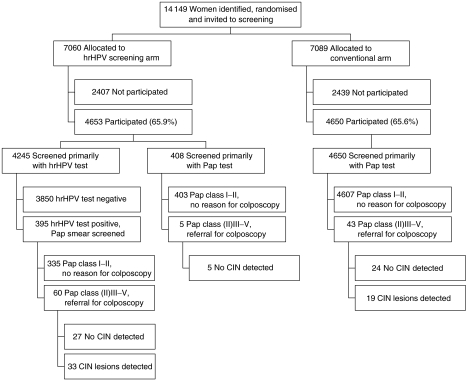
Randomised screening flow: participation, contamination, test positivity and confirmatory test results in the two arms.

As our aim was to measure the relative validity parameters for routine screening, we calculated the relative sensitivity (relative risk (RR)), specificity and positive predictive value (PPV) estimates for both arms by intention to screen. Relative risks of referral and histologically confirmed CIN were estimated in the hrHPV screening arm with 95% confidence intervals (CI) using the conventional screening arm as the reference (Miettinen & Nurminen, 1985). Specificity was defined as the proportion of the screening test negatives among those with no histologically confirmed lesion (including also those without biopsy). For the hrHPV screening arm, specificity was calculated with two definitions for test negativity: (1) primary screening test negative (see Figure 1 and Table 1) and (2) cytology triage negative (i.e. no referral for colposcopy). Similarly, PPVs were calculated for the hrHPV screening arm with two different cutoffs for test positivity: (1) primary screening test positive and (2) cytology triage positive (i.e. referral for colposcopy). Eventually, to clarify the significance of the observed differences in specificity estimates between study arms, we calculated 95% CIs for specificity with a binomial test.

## RESULTS

The number of women invited to hrHPV and conventional screening during the year 2003 was 14 149 altogether (7060 and 7089, respectively). Of the invited women 9303 attended (65.8%), 4653 (65.9%) in the hrHPV screening arm and 4650 (65.6%) in the conventional arm. The random allocation (intention to screen) was followed completely in the conventional arm, as all 4650 women had a cytological screening test. Instead, 408 women (8.8%) in the hrHPV screening arm were screened primarily with cytology – for the rest of the 4245 women, the primary hrHPV screening protocol was followed as intended (Figure 1). The mean age in the hrHPV arm was 45.8 years and that in the conventional arm 45.9, ranging in both arms from 30 to 60 years. Symptoms were reported equally often in the hrHPV and the conventional arm, in 10.2 and 10.1%, respectively.

Of the 4245 women tested for oncogenic HPV types, 395 (9.3%) turned out to be positive and, following the hrHPV screening protocol, cytology triage test was carried out for them. Based on the cytological test result, 60 hrHPV-positive women were eventually referred for colposcopy. Additional five referrals were made in the hrHPV arm among women screened primarily with cytology. In the conventional arm, a total of 43 women were referred (Table 1).

Compared with the conventional arm, clearly more colposcopy referrals were made in the hrHPV screening arm (RR 1.51, CI 95% 1.03–2.22). As a result of performed colposcopies, a total of 52 histologically confirmed precancerous lesions were detected within the study population: 33 in the hrHPV screening arm and 19 in the conventional arm. In the hrHPV screening arm, 17 of the lesions were low grade (CIN1), 10 moderate (CIN2) and six severe (CIN3+); in the conventional arm, they were eight, five and six, respectively. Relative risk point estimate for any CIN in the hrHPV screening arm was 1.74 (Table 2), which was not statistically significant, however. All lesions in the hrHPV arm were found among the hrHPV-positive women (see Figure 1).

In the hrHPV arm, specificity for the sole primary screening test was 92.1% for any lesion (CIN1+), 91.7% for moderate to severe lesions (CIN2+) and 91.5% for severe lesions (CIN3+). For the hrHPV screening with cytology triage, specificity estimates were 99.3, 98.9 and 98.7%, and for the conventional arm 99.6, 99.3 and 99.2%, respectively (Table 3).

For the sole primary screening test positivity, PPVs in the hrHPV screening arm were low, from 1.5 to 8.0% depending on the histological threshold. On the contrary, PPVs obtained with cytology triage (from 9.2 to 50.8%) were much more like those in the conventional arm (from 14.0 to 44.2%) (Table 4).

## DISCUSSION

In the current paper, we have reported the first-year results of primary hrHPV screening and of cytology triage protocol, incorporated in an existing cervical screening programme by randomised design. As a result, we observed a significant increase of 51% in the referrals of the hrHPV screening arm compared with the conventional arm. The detection rate at the level of mild and moderate dysplasia was two times higher in the hrHPV screening arm than in the conventional arm, but the same at the level of severe dysplasia. However, differences in detection rates were subject to considerable random variation and even more so with more stringent cutoff levels. Observed increase in cross-sectional relative sensitivity in hrHPV arm would have been acquired at the cost of substantial loss in specificity; however, with cytology triage the specificity was improved to the level of conventional cytology.

As the current results derive from a randomised population-based study analysed by intention to screen, most of the biases are avoided. Randomisation seems to be adequately performed, as no marked differences were seen between arms when comparing attendance rates, mean ages and reported symptoms. Although the attendance rate in the participating municipalities (65.8%) was lower than the mean in Finland (ca. 70%), it was about the level it had been in these municipalities during the previous years. Contamination rate in the intervention arm remained at moderate level (8.8%) and did not interfere with making the interpretations. Lack of blinding is the most potential cause of bias, as it might have affected the observed increase in referral and detection rates: the increase may result either from higher sensitivity of the hrHPV test or from overdiagnosis, that is, indolent lesions without potential of progression are detected as cytological, colposcopical and/or histological criteria are changed due to the knowledge on hrHPV status. However, as the study is run within routine programme, blinding was considered unnecessary. The reason of the observed increase will be verified in the subsequent follow-up of the women, together with the outcome evaluation.

Prior to our study, randomised trials on hrHPV testing in primary cervical screening have been conducted in Costa Rica (Guanacaste), USA (ASCUS-LSIL Triage Study, ALTS), The Netherlands (POBASCAM trial) and India. In the Guanacaste trial, sensitivity and specificity of the HC 2^©^ testing were compared to Pap testing using ASC-US cut point for colposcopy (Schiffman *et al*, 2000); however, the women tested for hrHPVs had multiple tests (a pelvic examination, conventional cytological test and LBC test, cervigrams) performed, which makes evaluating the outcome effectiveness of these tests impossible (The working group of IARC, 2005). Like in our study, in the ALTS and POBASCAM trial women were randomised to intervention and control groups, but in these trials hrHPV test (by HC 2^©^ in ALTS and by GP5+/6+ PCR immunoassay in POBASCAM) was used as a confirmatory test for cytological test, which is just the opposite to our study (Schiffman and Solomon, 2003; Bulkmans *et al*, 2004). The Indian trial, being most comparable to our study, resulted in specificity of HC 2^©^ test in detecting CIN2+ lesions ranging from 91.7 to 94.6%, and PPV from 9.1 to 16.7% – for the same cutoff in the current study, specificity estimate was 91.7% and PPV 4.0% (Sankaranarayanan *et al*, 2004). However, virtually all women in the Indian trial had histological confirmation, which did not happen in our study, as our ultimate aim is to evaluate the program effectiveness at the level of interval cancers; this kind of evaluation will not be possible in India, as the precancerous lesions of both test negative and test positive women were detected and treated.

Our results confirm the common finding that testing for hrHPVs is more sensitive but less specific than conventional cytological testing. Based on literature and our own pilot study results, this was what we had expected – being convinced that primary screening with sole hrHPV test would result in a substantial increase in the number of colposcopies (and moreover, increase in total costs and a possible increase in the reported adverse effects) in a country like Finland, where only clearly suspicious cytological findings (Pap class III–V) are traditionally considered a reason for colposcopy referral, we included a more specific confirmatory test, cytology triage, to our screening design (Nieminen *et al*, 2004). In addition, to not to increase the number of screening visits and dropouts in the hrHPV screening arm due to the two-staged confirmation, we decided that the sample for this cytology test was to be taken at the first visit together with hrHPV test sample. To reassure that the hrHPV test sample and endocervical subsample of the smear were taken exactly from the same location, only one brush sample was taken and used first for the smear and second for the hrHPV test.

Based on the first-year experience, cytology triage succeeded well in reducing the number of colposcopy referrals compared to the sole hrHPV test, which, from our point of view, seems justified as the primary screening method only if cytologists are not available in sufficient numbers: with moderate resources for cytological analysis, savings in the colposcopical resources may be earned. However, the hrHPV test itself is relatively expensive for screening purposes, especially when compared to conventional Pap smear; we have covered the excess costs of the hrHPV screening for the municipalities to keep the study ongoing. As lack of financial, infrastructural and manpower resources has prohibited the cytological screening programmes to start in low-resource countries, it is presumable that any form of hrHPV screening is too sophisticated and expensive to be feasible and effective in these countries, unless the hrHPV testing is made significantly cheaper and less dependent on hi-tech systems (Sankaranarayanan *et al*, 2004).

Interpretations on screening technologies have often been made on the basis of intermediate indicators instead of outcome (interval cancer rate) evaluation, and issues like overdiagnosis and other possible adverse effects of screening have not been widely discussed. Yet, sensitivity and effectiveness of the screening tests for cervical cancer can be reliably estimated only by means of interval cancer incidence for invasive disease. Without clear scientific evidence of better effectiveness of the screening programme with a new screening test, it is rational not to change a well-functioning existing programme: in ultimate outcome evaluation, it may turn out that the new screening modality is much more expensive to use, causes more adverse effects or anxiety and the results in terms of cancer incidence are hardly any better than with the old test. Interventions may produce different outcomes in effectiveness and efficiency in different countries, depending on their infrastructure and pre-existing screening programmes. Thus, we will continue the intake and the follow-up, and expand the population covered by hrHPV screening, as the results shown in this paper justify the further evaluation of this specific screening modality.

In conclusion, compared with conventional cytology, primary screening with hrHPV test results in increased cross-sectional relative sensitivity at the level of all positive lesions at the cost of substantial loss in specificity. With cytology triage the specificity improves to the level of conventional cytology.

## Figures and Tables

**Table 1:** Primary screening test results, colposcopy referrals and distribution of Pap class led into referral

	**hrHPV arm**				**Conventional arm**	
	**Primary hrHPV test**		**Primary Pap test**		**Primary Pap test**	
**Screening test result**	***n*=4245**	**%**	***n*=408**	**%**	***n*=4650**	**%**
Primary test negative	3850	90.7	403	98.8	4607	99.1
Primary test positive	395	9.3	5	1.2	43	0.9
*Colposcopy referral* a	60	1.4	5	1.2	43	0.9
Pap class II	3	0.1	0	0.0	3	0.1
Pap class III	55	1.3	5	1.2	36	0.8
Pap class IV	2	0.0	0	0	4	0.1
Pap class V	0	0.0	0	0	0	0.0

a In the hrHPV arm based on the result of cytological analysis carried out for the hrHPV positive (see study design for the protocol).

**Table 2:** Relative risk of colposcopy referral and histological findings in the hrHPV arm compared with the conventional arm

	**hrHPV arm**		**Conventional arm**			
**Colposcopy**	***n*=4653**	**%**	***n*=4650**	**%**	**RR**	**95% CI**
Referral	65	1.40	43	0.92	1.51	1.03–2.22
*Any CIN*	33	0.71	19	0.41	1.74	0.99–3.05
*CIN1*	17	0.37	8	0.17	2.12	0.92–4.92
CIN2	10	0.21	5	0.11	2.00	0.68–5.84
CIN3+	6	0.13	6	0.13	1.00	0.32–3.10

RR=relative risk; CI=confidence interval.

**Table 3:** Specificity of the sole hrHPV test and hrHPV screening with cytology triage in comparison with conventional screening calculated with three histological cutoffs

**Cutoff**	**No histological confirmation or negative at cutoff (*n*)**	**Screening test negative (*n*)**	**Specificity (%)**	**95% CI (%)**
*hrHPV arm, sole hrHPV test (n*=*4653)*				
CIN1+	4620	4253	92.1	91.2–92.8^a^
CIN2+	4637	4253	91.7	90.9–92.5^a^
CIN3+	4647	4253	91.5	90.7–92.3^a^
				
*hrHPV arm, cytology triage (n*=*4653)*				
CIN1+	4620	4588	99.3	99.0–99.5
CIN2+	4637	4588	98.9	98.6–99.2
CIN3+	4647	4588	98.7	98.4–99.0^b^
				
*Conventional arm (n*=*4650)*				
CIN1+	4626	4607	99.6	99.4–99.8
CIN2+	4639	4607	99.3	99.0–99.5
CIN3+	4644	4607	99.2	98.9–99.4

hrHPV=high-risk human papillomavirus; CI=confidence interval.

a In comparison to the specificity of the conventional arm with the same histological cutoff, *P*<0.0001.

b In comparison to the specificity of the conventional arm with the same histological cutoff, *P*<0.05.

**Table 4:** Positive predictive value of the sole hrHPV test and hrHPV screening with cytology triage in comparison with conventional screening calculated with three histological cutoffs

**Cutoff**	**Screening test positive (*n*)**	**Positive histology at cutoff (*n*)**	**PPV (%)**
*hrHPV arm, sole hrHPV test (n*=*4653)*			
CIN1+	400	33	8.0^a^
CIN2+	400	16	4.0^a^
CIN3+	400	6	1.5^a^
			
*hrHPV arm, cytology triage (n*=*4653)*			
CIN1+	65	33	50.8
CIN2+	65	16	24.6
CIN3+	65	6	9.2
			
*Conventional arm (n*=*4650)*			
CIN1+	43	19	44.2
CIN2+	43	11	25.6
CIN3+	43	6	14.0

hrHPV=high-risk human papillomavirus; PPV=positive predictive value.

a In comparison to the specificity of the conventional arm with the same histological cutoff, *P*<0.0001.
